# The Analysis of Green Areas’ Accessibility in Comparison with Statistical Data in Poland

**DOI:** 10.3390/ijerph17124492

**Published:** 2020-06-22

**Authors:** Joanna Wysmułek, Maria Hełdak, Anatolii Kucher

**Affiliations:** 1 Institute of Spatial Management, Wrocław University of Environmental and Life Sciences, 55 Grunwaldzka Street, 50357 Wrocław, Poland; maria.heldak@upwr.edu.pl; 2 V. N. Karazin Kharkiv National University, Svobody Square, 6, 61022 Kharkiv, Ukraine; anatoliy_kucher@ukr.net

**Keywords:** natural green space accessibility, public green space, ANGSt method, spatial policy, environmental planning

## Abstract

The study discusses the problem of public green areas’ accessibility for the residents of large cities in Poland. The purpose of the research is to assess the possibility of applying the British Accessible Natural Greenspace Standard (ANGSt) method in determining the amount of natural green space available to residents in Polish conditions including, in particular, the assessment of accessibility using data collected by the Central Statistical Office and the verification of results based on detailed research. The identification of green areas for 18 voivodeship cities in Poland was prepared using the GIS programme, taking into account public green space, provided for general access and free of change. The verification of the ANGSt method consisted of mapping spatial barriers extending the route of access either on foot or by roads as well as closed private areas. The conducted research revealed that, after taking into account the access routes to selected areas, the distance to public green areas increased, on average, from 50 m in the smallest cities (Gorzów Wielkopolski and Olsztyn) to as much as 450 m in Warszawa. A detailed analysis showed that the discussed accessibility was reduced, on average, by almost 10% for the residents of the analysed cities. It was also found that the introduced barriers did not affect the accessibility of more distant, larger green space areas.

## 1. Introduction

The quality of life in a city is affected, among other things, by physical, social, environmental and economic characteristics of a given space [1,2]. The places where residents can actively and passively rest include, e.g., sports facilities (swimming pools, fitness clubs, sports halls), cultural institutions (cinemas, theatres, operas, museums) and urban greenery (parks, green squares, gardens, street and residential area greenery, forests, green protected areas). The choice of a place for a short rest during the day and the type of relaxation often depend on the distance to cover and on certain natural factors (e.g., the presence of lawns, trees, shrubs, surface waters, varied terrain) and also non-natural ones (parking lots, paths, places for active recreation, playgrounds, picnic areas, elements of small architecture, historical buildings and educational facilities) [3].

Scientific research has shown that green areas provide people with a wide range of advantages that contribute to their health and well-being, as well as improve their quality of life [4,5,6,7]. They function as the green lungs in cities by absorbing pollution [8,9], thus providing people with space to enjoy nature and take advantage of it in the stressful moments of modern life [10,11,12].

Urban green areas also perform aesthetic, environmental, health and recreational functions. All these qualities show that greenery is an important element of sustainable urban development.

The positive contribution made by green areas in people’s lives increases the demand for more reserves and access to green areas both in cities and around them [13]. It was also found that residing close to natural green space results in health benefits [2,14,15,16], in advantages for the environment [17,18] and also in economic profits [19]. The aforementioned compilation of research highlights numerous advantages of greenery and the way in which accessing it brings benefits to both public health and social well-being. In order to emphasize its value and advantages, urban standards regarding the arrangements for open areas were defined in Poland in the 1970s (e.g., within 800 m from the place of residence a recreational park, covering the area of 2 ha, should be provided).

Unfortunately, the set standards were not met, and in the 1990s of the 20th century and in the years to follow, the undeveloped areas were designated for commercial purposes [20].

Currently, no central regulations oblige designers of local plans to provide public green areas and recreation within housing structures. The applicable law applies only to the area covered by the investment, stating that the minimum 25% of its area should be a biologically active area, if a different percentage does not result from the findings of the local zoning regulations [21]. This creates a large field for over-interpretation of regulations.

Organizational units in most countries use indicators related to the surface share of greenery when planning spatial development strategies for cities. In practice, they most often use data published by the Central Statistical Office. However, it is limited to taking into account only a few categories of greenery. It does not take into account, among other things, the distance of inhabitants’ residences from green areas. The actual share of areas of natural and social significance may be significantly different from the values published by the Central Statistical Office, depending on the land use structure. Reliance only on these data can have a disastrous effect on local government administration units when undertaking program and strategic activities related to urban development.

Many researchers indicate the great importance of urban green planting for the development of residential housing [22,23,24]. The current construction law is limited to providing guidelines regarding the presence of biologically active areas within a given investment in accordance with the local land development plan. There are no guidelines requiring the establishment of large-area greenery facilities. It results in limiting the green areas in the newly developed residential housing areas to the minimum and not using greenery with a significant abundance of leaves [3,20]. It also reduces their recreational potential, approached as the capacity of the environment to create conditions providing rest and physical and mental recovery to people [4,25,26]. An additional reason for the recreational potential decline is the chaotic way in which spatial development takes place [27]. Charles Landry [28] (p. 252) believes that “urban planning is suffocated by the absence of allegedly unprofitable investments in public infrastructure”. Current inclusion of greenery, as areas in the spatial design of a modern city centre, becomes increasingly difficult to implement because there are either no free areas or they are too valuable for this type of investment. The green areas available in Polish cities are continuously shrinking, which concerns both the city centres and residential districts, public and semi-private spaces (e.g., as a result of the liquidation of front gardens, functioning in residential districts in the previous century, in favour of parking spaces). Green areas disappear, similarly to their usage methods [29].

The research covered the selected, publicly accessible, recreational and free of charge green spaces. The following green areas were distinguished: parks, squares, lawns, residential green spaces, street greenery and municipal woodland. The definition of urban greenery in accordance with the dictionary of basic land use and spatial planning terms has been adopted in the article, i.e., “green spaces in cities, covered with vegetation presenting ecological, protective, recreational and aesthetic features”. Urban greenery occurs in the form of parks, squares, gardens, isolation and protective greenery; it occurs in inner-city areas, housing, leisure, industrial and suburban areas; it creates a band, wedge, radial, annular, irregular, compact or distracted system in the city; and it can be part of the city’s natural system and fragments of the ecological system of protected areas [30] (p. 145).

Moreover, according to Piątkowska K. [31] (pp. 37–38), green areas are open and are consciously composed and multifunctional, including, inter alia, in the protection and shaping of the climate and the environment, as well as in the scope of social and service functions provided for the residents. They can occur both in the city, as urban green areas and recreation, as well as in areas associated with weekend and periodic rest, with the rural settlement network or in industrial areas.

According to the explanation from Bartosiewicz and Brzywczy-Kunińska [32] (pp. 11–12), green areas are shaped according to the plans for the development of cities and housing estates. In addition, there are public green territories available to everyone, such as parks and green areas, but also greenery closed or intended for a limited group of people, for example, gardens in schools, hospitals, factories or plots. Based on the diverse beneficial influence of green areas on urban units, many studies have focused on analysing access to natural green spaces. The importance of accessibility and access to green areas was analysed taking into account two perspectives: accessibility in terms of travel and distance and also the perception of such access. From a distance perspective, accessibility is defined as “the ease with which activities at one place may be reached from another via a particular travel mode” [33] (p. 105). Therefore, a number of studies have adopted geographic information systems (GIS) to analyse the accessibility of green areas based on distance, travel time and costs. For example, Neuvonen et al. [34] analysed the relationship between access to natural green areas and the frequency of visits. Oh and Jeong [35] studied the accessibility of pedestrians to city parks. There is also a large number of studies researching the accessibility of green space using both distance and travel time and focusing on equal access to green areas in different communities, supporting the concept of environmental justice [36,37,38,39,40,41,42].

There are many methods for analysing access to green areas. The methods used in the discussion presented in this study on the research results include, e.g., the method used by Feltynowski et al. in Poland [43]. It provided the comparison of data from five publicly available sources: (1) public statistics, (2) the national land surveying agency, (3) satellite imagery (Landsat data), (4) the Urban Atlas, and (5) the Open Street Map. The results reveal large differences in the total amount of urban green spaces in the cities as depicted in different datasets. Other studies cited in the discussion are the ones carried out in Romania by Badiu D.L et al. [44]. She emphasizes that the most used quantitative indicator to assess urban green infrastructure is urban green space (UGS) per capita. In order to address the problem of urban sustainable development, 26 m^2^ were adopted per resident in all cities. Aerial photos were taken for 38 cities; next, the data were compared with three other databases (National Institute of Statistics, Environmental Protection Agencies and Urban Atlas) to check for differences. In addition, the significant differences between the surface of UGS reported by the administrative offices and those resulting from the spatial analysis were found. In China, Feng et al. examined the relationship between urban park accessibility and population distribution at different administrative levels [45].

The presented article focuses primarily on the assessment of the availability of natural green areas in Polish provincial cities. It takes into account the application of the British ANGSt (Accessible Natural Greenspace Standard) method used to determine the amount of natural green available to residents, adopting a modification to Polish conditions. It also verifies the results obtained with indicators regarding green areas published by the Central Statistical Office. The process of performing ANGSt includes determining the research area and relevant data sources, filtering data to meet the adopted criteria for available green areas. Mapping works are performed using the geographic information system (GIS) software. As the work progressed, it was also decided to carry out modified analyses imposing spatial barriers to the model.

The following research hypotheses were adopted in the study:

1. Residents of large cities have difficulty accessing the generally accessible public green spaces of 2 ha in area and more, located within 300 m distance from a housing estate.
2. The data collected by the Central Statistical Office are insufficient for planning and designating green areas in the urban space and should be supported by scientific research.

Nowadays, both in legal regulations and in the literature of the subject, there is no unambiguous interpretation for the areas covered with vegetation located in the city.

## 2. Materials and Methods

### 2.1. Study Area

**Figure 1:**
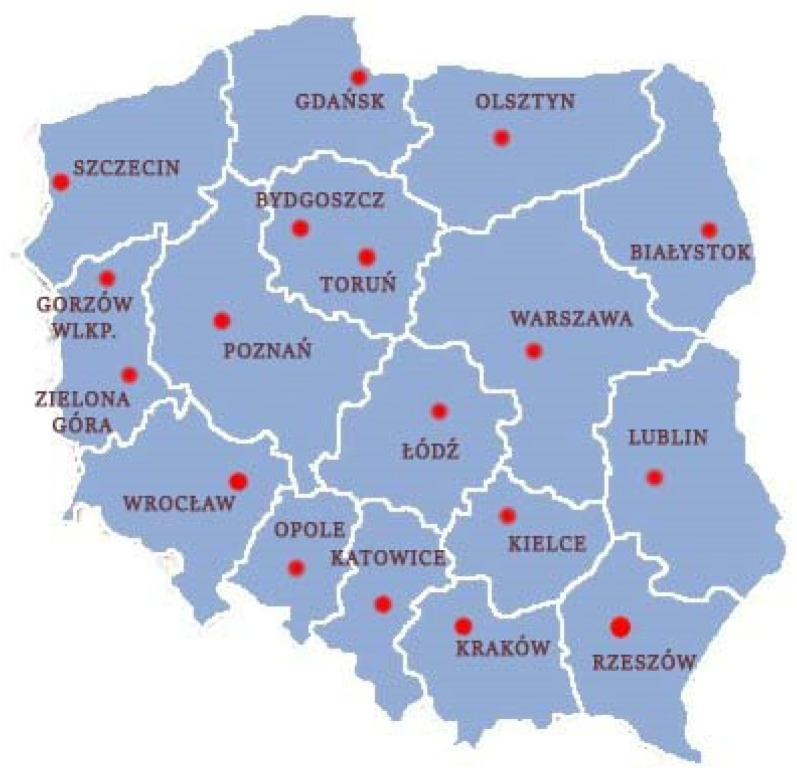
Location of study area with boundaries of voivodships.

The presented research is focused on the assessment of the amount of greenery available in terms of recreation for the residents of Polish voivodeship cities. Since 1999, the country has been divided into 16 voivodeships. In two regions, Lubusz and Kuyavian-Pomeranian, the seat of the marshal and governor was divided into two cities. Thus, the research covers 18 voivodeship capitals (Figure 1). The boundary of the studied areas was the 10 km buffer from the administrative borders of the cities.

Table 1 also presents the indicator of public green area per inhabitant (1 January 2019).

### 2.2. Research Methods

The applied research method followed the steps listed below:

- the identification of the research problem and the selection of the research area;
- the subject literature analysis;
- the selection of public green areas’ categories constituting the subject of conducted analyses;
- preparing statistical data on natural green areas in 18 capitals of voivodeships in Poland;
- spatial identification of natural green areas in individual voivodeship cities using the GIS programme;
- mapping accessibility buffers using the ANGSt method, following prior identification of housing estates;
- the obtained results verification against the data retrieved from the Central Statistical Office;
- the verification of assessment results regarding the accessibility to public green areas using the ANGSt method based on the identification of spatial barriers;
- re-assessment of the accessibility to public green areas;
- and conclusions.

Due to the low popularity of recreational activities in cemeteries, as well as their variety in terms of vegetation, and also a fairly low percentage and qualitative diversity in relation to total green zone, it was decided to skip this component.

Family allotment gardens were also left out due to their limited, in most cases, accessibility for the general public (the majority of gardens are closed and only the owners of individual gardens have access to them). Pastures, arable land, meadows or undeveloped riverside and post-industrial areas were not taken into account either.

The subject of the analyses was verified in terms of the green area defined in the Nature Conservation Act [47]: “green space stands for the arranged areas along with technical infrastructure and buildings functionally connected with them, covered with vegetation, performing public functions and in particular: parks, lawns, promenades, boulevards, botanical and zoological gardens, children game parks and historic gardens, cemeteries, greenery along the roads in built-up areas, green squares, historic fortifications, constructions, storage sites, airports, railway stations and industrial facilities”. The verification also covered the accessibility of selected areas for residents.

After the units were separated for further research, a compilation of statistical data made available by the Central Statistical Office for green areas in 18 voivodeship cities in Poland was carried out. The availability of data and the method of interpretation of green areas were the basis for selecting the data source: “Green urban areas consist of parks, urban forests, green squares, green residential spaces, street greenery, zoological and botanical gardens, as well as cemeteries. The key to choosing this type of green areas is that they are managed by the municipal authorities” [43] (p. 61). In order to determine the tendency for the transformation of green areas, the values from three different years, i.e., 2004, 2010 and 2018, were compared. The areas of cities from the same periods were also compared.

The data provided by the Central Statistical Office are available on the website www.stat.gov.pl. It collects and provides information on, among others: residents, the environment, living conditions, education, culture, work, finance, economy and natural goods. City authorities and business intelligence services often use this information. Their advantage is high data availability. Thanks to this, analyses can be conducted on full material collections. Another advantage is the lack of influence of third parties on the data creation process. On the other hand, the main disadvantages include the reliability and method of obtaining data.

The next stage of the research was the spatial identification of green areas in individual voivodeship cities in Poland. Taking into account a rather limited concept of public green space represented by public statistics and the discrepancies in the occurrence of green areas in cities [43], municipal spatial information systems and the topographic database were used for the purpose of mapping green areas. In order to verify green areas more accurately, the cities were divided into smaller units (specified individually for each centre). The minimum mapping unit was 50 m. In case of doubts regarding free accessibility, the areas were verified individually by searching for information on the local authorities’ websites. Next, the particular layers were mapped in the GIS and green areas were selected using the aforementioned guidelines. The underlay for each city was then buffered using the recommendations provided in the ANGSt method. This technique was developed in 1998 to study the extent to which the population living in the southeastern part of Great Britain has access to public green areas.

The adapted method determined the minimum area of greenery to be 2 ha. According to many researchers [48,49], expanses with smaller areas are insufficient to provide adults with adequate support for physical activity. The size of green areas can also slow down the phenomenon of appropriation of land by one group of people [50,51,52].

The following standards specify the distance between the place of residence and green areas presenting particular surfaces, in accordance with the ANGSt method [53]:

- No person should live more than 300 m from their nearest area of accessible natural green space of at least 2 ha in size.
- There should be at least one 20 ha accessible natural green area within 2 km from home.
- There should be one accessible natural green zone 100 ha site within 5 km.
- There should be one accessible natural green space 500 ha within 10 km.

Following the above recommendations, green areas of less than 2 ha in size were excluded from the analysis. In addition to the areas within the administrative borders of individual cities, a 10 km buffer around them was also taken into account (maximum value for the ANGSt model). Buffers were deleted using ArcGIS. Due to the concentration of the objects, Euclidean buffers were used. Considering the fact that a city constitutes the subject of research, the earlier defined green areas were adopted as natural green areas.

Then, using the data contained in the spatial information system of provincial cities, a layer presenting the estate was applied. Prior distinguishing smaller units, for which it was easier to determine the number of households, turned out to be helpful. The population was counted using an average statistical size of a household—3 people in a dwelling [46]. On this basis, the percentage of the population having access to individual green areas was calculated. The next step was the verification of the obtained results against the data retrieved from the Central Statistical Office [46]. The final research stage consisted of mapping spatial barriers. Major line infrastructures (railways, highways, roads with more than 3 lanes, bridges, and rivers) as well as private premises were mapped using the topographic database. It was decided that in the city centre, where pedestrian crossings are quite densely designated, the occurring barriers would not be taken into account, except for railway lines and waterways [54]. The research was finalised with reassessing residents’ accessibility to the generally accessible green areas.

The presented results were divided into three sections. The first of them verifies the data retrieved from the Central Statistical Office based on the areas selected for further analyses. The second focuses on the results of the ANGSt method application for the selected cities and analyses how population levels in individual cities relate to them. The third section contains maps summarizing the generally accessible green areas with the additionally designated spatial barriers.

## 3. Results

### 3.1. Descriptive Statistics

Prior to the spatial identification of green areas in individual cities, the general characteristics of cities were determined based on the data from the Central Statistical Office. The ratio of the area of generally accessible green spaces (the area of municipal forests, parks, lawns, residential green spaces and street greenery was summed up) against the area of particular cities was calculated (Table 2).

**Figure 2:**
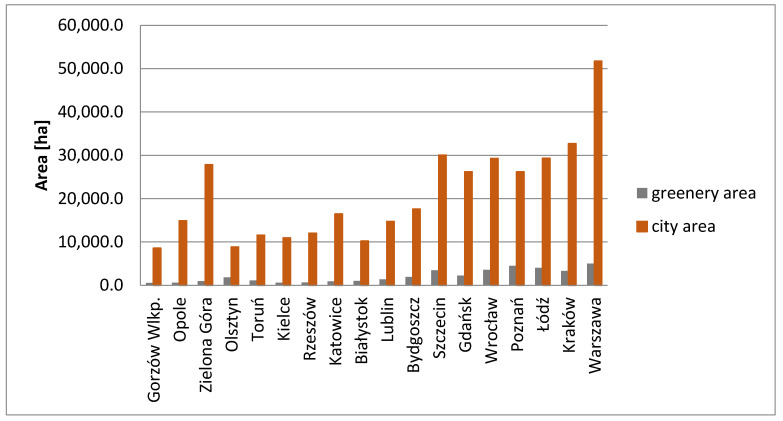
The area of cities and green spaces by the increasing number of residents.

The above table shows changes taking place in the share of the generally accessible green areas over the period of 13 years. They can result from alterations in the interpretation of urban green space as well as functional transformations of the areas covering both the creation of new green areas and changing the existing ones into either residential housing or service and industrial buildings. No correlation was identified between the size of individual cities, the number of residents and the occupied green area (Figure 2).

Furthermore, the areas selected for further analysis on the basis of urban geoportals did not show any connection between the areas of cities and those of the generally accessible green zones located in their boundaries.

### 3.2. Accessible Natural Greenspace Standard

Following the standards developed based on the British ANGSt method, the residents’ distances to green areas of particular surfaces in 18 cities were presented (Table 3). After compiling four layers for each city, the percentage of residents for which all criteria were met (column “All of the ANGSt Requirements Met”) was obtained.

The above table shows that the residents of Kielce, Szczecin and Opole have the relatively largest access to green spaces of 2 ha and more within a 300 m radius. In turn, the smallest access refers to the residents of Białystok, Katowice and Rzeszów. The obtained results indicate that the situation of the residents in the analysed cities does not seem to be good. In the largest Polish cities, such as Warszawa, Gdańsk, Wrocław and Poznań, the comfort of using the generally accessible green spaces within a 300 m radius and offering 2 ha of area is available for only about 20% of the residents.

These studies, however, require more detailed analyses addressing the access to smaller squares and playgrounds, which has been indicated as the subject matter of the planned research.

**Figure 3:**
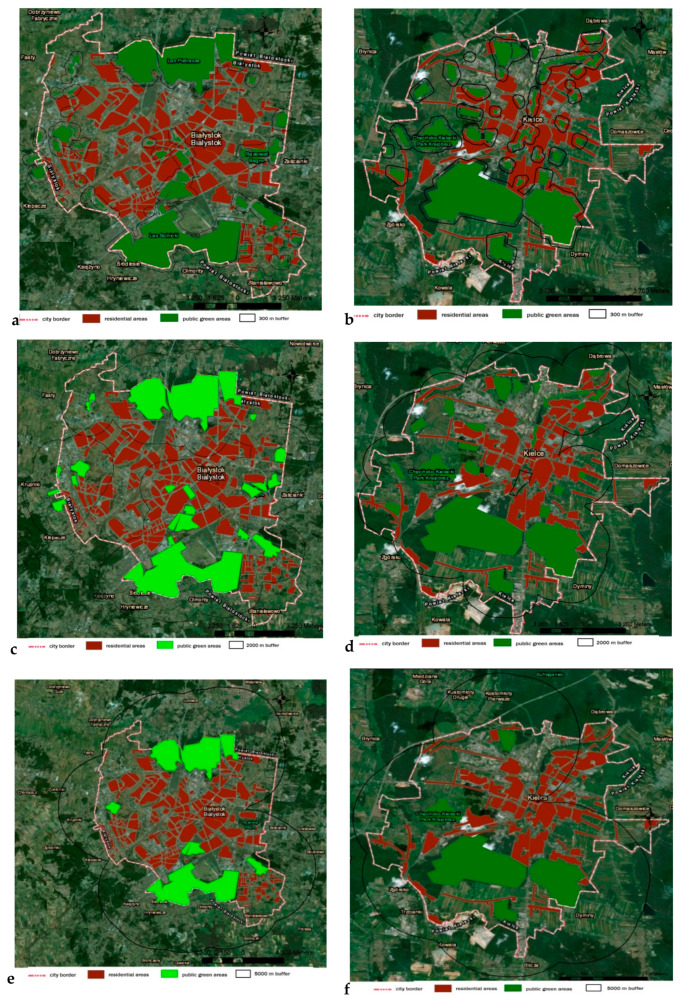
Access of residents to green spaces: (**a**) 300 m buffer in Białystok, (**b**) 300 m buffer in Kielce, (**c**) 2 km buffer in Białystok, (**d**) 2 km buffer in Kielce, (**e**) 5 km buffer in Białystok, and (**f**) 5 km buffer in Kielce.

The most noteworthy research result is the absence of accessibility to green areas closest to residential estates. In the analysed cities, the accessibility of areas covered by the generally accessible vegetation presenting the surface of 2 ha, located in the immediate vicinity (300 m), ranges from 9% to 38% of the residents’ number. Figure 3 shows the availability of greenery for residents based on the examples of Białystok and Kielce, respectively the largest and smallest percentages of access to green areas within 300 m.

The conducted studies have also shown that the accessibility of greenery does not depend on the city size. The surface of the areas featuring natural values does not show any correlation with the number of residents. Furthermore, the statistical data, showing the ratio of green areas against the size of individual cities, do not affect residents’ accessibility to green areas, primarily the ones in the immediate vicinity (Table 4). After comparing the data in the table, it can be seen that cities with a higher ratio of green areas to their entire area do not have greater availability of green areas in the closest distance for residents. After analysing the types of areas found in individual cities, it was noticed that the higher ratio of green areas to the city surface is often due to the distribution of greenery in the peripheral areas of the city—most often, they are large forest areas. However, they do not translate into an increase in available green areas in the immediate vicinity of residential areas. As shown below (Table 4), e.g., Kielce, despite one of the smaller relations of green areas to the surface of the whole city, has the highest access of green areas in the immediate vicinity of residential areas.

### 3.3. Spatial Barriers

The last step consisted of mapping spatial barriers. After taking into account the access routes to the selected areas, it occurred that the average distance was extended from 50 m in the smallest cities (Gorzów Wielkopolski and Olsztyn) to as much as 450 m in Warszawa, resulting in reduction of accessibility for residents by 9% (see Table 5).

To compare the data collected before and after the barriers were applied, tests were performed to see if there was a statistically significant change. Nonparametric tests were used. To compare two paired groups for variables that do not have a normal distribution, the Wilcoxon signed-rank test and the sign test were used. The calculations were made in the STATISTICA program.

The Wilcoxon test consists in ranking differences in measurements for subsequent observations; the differences between measurement 1 and 2 are calculated. Then, these differences are ranked, i.e., the results obtained are ranged from smallest to highest and given subsequent ranks. Then, the sum of ranks is counted for differences that were negative and for differences that were positive (results with no differences are not significant). Then selected the sum (negative or positive differences) that was greater, and this result is the result of the Wilcoxon test (up to 25 observations). Then, the units are converted to *Z* values: Wilcoxon test result (i.e., the highest sum of ranks), the average divided by the standard deviation. This result is checked in the normal distribution tables [55].

*H*_0_—The results of trial 1 (access at a distance of 300 m) and trial 2 (access at a distance of 300 m with barriers) are the same/statistically insignificant.

*H*_1_—The results of trial 1 (access at a distance of 300 m) and trial 2 (access at a distance of 300 m with barriers) are statistically significant.

Values read in the program: *Z* value: 3.724; p=0.00020<0.05∝

Results in the sign test:

(1) Z=4.01 p=0.00006<0.05∝

∝−level of significance.

The tests before and after applying the barriers differ from each other with statistical significance.

Introducing barriers did not affect the accessibility of further located larger green areas.

## 4. Discussion

The selected type of analysis can never provide all the answers to the complex range of problems with access to green areas. One of the important reasons for undertaking the work was to draw attention to the problem of urban planning.

The need of green areas’ accessibility is discussed with increasing frequency, e.g., [34,36,40,41,56,57,58,59]. The problems are the set of data regarding green areas, the coherence of such data and the absence of general classification in terms of green space. The data collected by the Central Statistical Office depend on, e.g., changes in land ownership or quite general and diverse interpretation of the problem over the years. It poses a major challenge for urban spatial planning and management of green areas [44]. Raw data not subjected to an in-depth analysis can present a false image of the existing situation. In addition, the interpretation of the term “green areas” in different cities differs, even if they are to comply with the general definitions used by the Central Statistical Office.

The conducted research placed emphasis only on the generally accessible areas with surface exceeding 2 ha, in accordance with the ANGSt standards. It is, however, necessary to pay attention to the fact that, while planning and managing urban space, such areas as arable land or private and informal green zones should be taken into account. They can constitute an additional place for meetings and recreation for a wider population, and can also play the role of an ecological passage. Moreover, smaller areas, e.g., the increasingly popular pocket parks in built-up areas, can represent a good tool in creating new public spaces [23,60].

The additional problem in conducting the discussed research was the absence of a unified classification covering green fields for all voivodeship cities. In some of them, the inventory data concerning urban green areas were missing. This means that there is a need for introducing land use classes or land cover as the indicators of what green space stands for and, thus, how to combine it with the rest of ecosystem services. However, as shown by the latest comparative studies carried out by Kremer et al. [61], due to the differences in urban morphology and its heterogeneity, even such an approach has its own limitations. As a result, the research that was conducted provides vital information on urban ecosystem services only in these areas where local or regional measurements were performed [62].

In addition, spatial data from public statistics are collected only for a municipality or an equivalent unit and cannot be divided into smaller administrative units (e.g., districts or housing estates), whereas green space data are primarily important in a local scale. Other researchers also compared various information sources about green areas in urban space and came to similar conclusions. For example, Feltynowski et al. [43] compared satellite imagery with the Lodz public statistics, the national land surveying agency, Open Street Map and the Urban Atlas, and identified significant differences between them. They noticed that the most comprehensive set of data from the country is the set from a geodetic agency, which shows that greenery constitutes 61.2% of the city’s area. In their research, however, they did not take into account the accessibility to green areas by residents. Similar studies were conducted in Romania, although, in the case of those cities, official statistics overestimated green areas in comparison to aerial photographs [44].

Research studies carried out, among others, in China also show that the availability of public parks for city dwellers is often unevenly distributed. Analyses conducted by Feng et al. [45] were carried out in parks in Beijing, and the relationship between the availability of city parks and population distribution at various administrative levels was analysed. The results of their research showed that the availability of city parks is different at the administrative level and that places with more city parks usually have more accessibility. Zhang et al. [63] also conducted research related to equal access of residents to green areas. The results obtained show, similarly to those presented in this article, that the current planning of urban public greenery using only the ratio of public greenery to urban land and public greenery per inhabitant is insufficient. Currently, planning of open space areas in Polish cities is based on a narrow classification used for public statistics purposes, covering formal green areas managed by the authorities. As in the case of Chinese cities, in Poland, there is also a lack of information about recreational areas covering smaller ranges (districts or housing estates). It poses a barrier for green areas’ planning in urban infrastructure and making them accessible to residents. It also introduces false information about the anticipated needs, e.g., large forest areas located on the outskirts of cities increase the green areas’ ratio, although they do not fully affect their share in the accessibility among residents.

The authors concluded that a more consistent approach to green space qualification could turn out to be helpful for reporting purposes throughout the entire European Union—particularly, unification of the green area terminology and the approach to private, semi-private and open spaces. In order to facilitate management and research in the field of green zones, as well as further development of urban areas, data sets should provide information who exactly is responsible for each space management. Predominantly, various municipal institutions are interested in those green regions for which they are formally responsible and, in the situation of poor cooperation between various stakeholders, they neglect other green areas and connections between them [43,64].

Another question asked by the authors referred to the selection of size and distance from green areas. Within the framework of the research continuation, it is worth reversing the question—what is the distance from residential areas to green areas and what is their surface size? The research should also be extended by the verification of human behaviour—are the residents willing to spend time in the selected green units?

The ANGSt model does not require assessing the quality components which the conducted research did not provide information about, e.g., such facilities as car parks or access for persons with disabilities, as well as proper markings and promotion of the areas. It mainly applies to the areas distant from the place of residence by 5 km and more. The quality of landscape or the sense of peace and quiet were not taken into account either. Yet another limitation of this study, at least in terms of the population pressure model, is the fact that it does not consider the population visiting a given site but not residing in the analysed region. In terms of the ANGSt model, this fact is irrelevant, as its requirements were established for the green space at the local level (i.e., up to 10 km from the place of residence). However, the protected landscapes, and national parks in particular, will always have visitors, often in large numbers, coming from outside the distance provided for in the ANGSt model, which can negatively affect the quality of the user experience and also increase the likelihood of harmful consequences resulting from a high degree of visitor pressure.

## 5. Conclusions

The conducted research allowed for formulating the following conclusions:

- The accessibility assessment of the selected large cities’ residents in Poland to green areas of 2 ha and more within a 300 m radius does not seem to be good. Based on the applied Accessible Natural Greenspace Standard (ANGSt) method, it was found to range between 6% and 38%, with a median of 20.5%. This confirms the first hypothesis—that the residents of large cities have difficulty accessing public green areas of general public accessibility with an area of 2 ha and more, located within 300 m distance from a housing estate.
- Detailed analysis based on the Accessible Natural Greenspace Standard (ANGSt) method in Poland have revealed some of its shortcomings. Namely, determining the buffers of the required distance from the areas of public green zone, without including spatial barriers, distorts the actual route of reaching them; the method should be modified to assess the distance from green areas and take into account existing spatial barriers. In the course of the conducted analyses, it has been found that the route covered between “the place of residence and the green space” can, in fact, be much longer. This reduces the quality of life measured by the distance from green spaces of varying area and, additionally, reduces the share of residents’ access to the publicly accessible green areas.
- The research revealed changes in the assessment of green areas’ accessibility in the case of the ANGSt method modification, in particular, regarding the green space located in the close proximity of residential areas (2 ha surface size, at a distance of 300 m). The detailed analysis of voivodeship capitals, in terms of the discussed problem, did not present favourable results (the route extension from 50 m in Gorzów Wielkopolski and Olsztyn, to 450 m in Warszawa, taking into account spatial barriers, was recorded). At the same time, no changes in the assessment of accessibility to large-scale urban green areas (500 ha surface size at a distance of 10 km) were observed. The method modification revealed some of its shortcomings; spatial barriers (rivers, railways, and communication arteries) are frequently impossible to get through for a mother with a child or a senior citizen. In turn, extending the route can result in making such distance impossible to cover on foot.
- The analysis of existing legal acts as well as standards and design guidelines for housing estates confirms the first thesis, i.e., in Poland, there are no standards defining the distance to the generally accessible green areas to be met per one resident. Taking into account the accessibility of green areas, especially the ones in the immediate vicinity, is important for further discussions on the delimitation of green areas in cities, primarily from the perspective of urban planning and management of urban green areas in order to provide ecosystem services and ensure adequate access to these places. Certain works have been undertaken many times; however, due to the absence of possibility for their implementation, they usually remained within the sphere of projects.
- The problems encountered during the research that was conducted in finding the reliable statistical data also confirmed the second thesis. The data collected by the Central Statistical Office are insufficient for urban green space planning and management. They should be supplemented by scientific research for the proper development of urban areas. The problem refers to, e.g., the area land classified in the Land and Property Register as forest and woodland in cities is not always used for recreational greenery purposes. The difference between the registered status and the actual development is often significant. An ongoing update of the land and property records by means of site visits should be performed. Scientific research introduces some modernization in space management; therefore, simulations and solutions preceded by the respective analyses of variants aimed at improving access to the organized forms of recreation would certainly turn out crucial in this case.

The research results can be used to determine priorities in the analysed regions as well as identify the key areas lacking accessibility to green spaces and take them into account in urban spatial policy.

**Planned research**: The research should be continued and focus on the behaviour of residents and their willingness to take advantage of green space, and also on determining whether and at what distance from the place of residence the areas smaller than 2 ha in size (not covered by the presented research) are located. In addition, the access to green spaces targeted at different age groups remains an important problem for future research. The authors will conduct relevant analyses taking into account the needs of younger children, older children, young people, adults and older people within smaller than 2 ha of generally accessible green areas. Following the above, they intend to analyse access to playgrounds with various equipment, sports fields, walking areas, open air gyms, walking paths, etc.

## Figures and Tables

**Table 1:** The percentage of cemeteries’ area against the area of public green space and indicator of green area in individual voivodeship cities in Poland.

City	Public Green Space [ha]		Cemeteries’ Area [%]	Indicator of Green Area per Inhabitant [m^2^/os]	
	2010	2018	2010	2018	2018
Białystok	896.9	1041.8	9.9	9.0	35.51
Bydgoszcz	1911.3	1927.1	5.2	4.9	54.44
Gdańsk	2092.0	2249.2	4.4	4.2	38.63
Gorzów Wlkp.	484.3	533.4	7.4	7.1	43.67
Katowice	1228.1	919.9	6.3	8.4	30.42
Kielce	584.3	593.3	7.5	8.0	30.28
Kraków	3230.2	3297.2	4.3	4.1	42.97
Lublin	1183.0	1380.6	6.4	5.5	40.36
Łódź	3644.3	4044.6	6.2	5.6	58.20
Olsztyn	2127.9	1851.3	3.8	4.4	29.29
Opole	643.0	585.1	7.0	9.3	49.46
Poznań	3739.6	4475.9	6.7	5.6	81.14
Rzeszów	658.2	636.1	7.1	8.6	34.59
Szczecin	3316.6	3497.0	5.6	6.1	86.46
Toruń	1008.9	1135.3	8.4	7.5	56.92
Warszawa	4634.6	4988.3	8.0	7.7	28.26
Wrocław	2930.6	3580.5	4.9	3.9	56.65
Zielona Góra	898.1	946.4	3.2	7.4	68.02

Based on: Local Data Bank from the Central Statistical Office [46].

**Table 2:** The area of generally accessible green space against the area of individual voivodeship cities in Poland.

City	Greenery [ha]			The Ratio of the Area of Generally Accessible Green Spaces to the City’s Surface [%]		
	2004	2010	2018	2004	2010	2018
Białystok	727.3	896.9	1041.8	8	8	9
Bydgoszcz	1662.7	1911.3	1927.1	10	10	10
Gdańsk	1789.0	2092.0	2249.2	7	8	8
Gorzów Wlkp.	525.5	484.3	533.4	6	5	6
Katowice	1266.1	1228.1	919.9	8	7	5
Kielce	483.0	584.3	593.3	4	5	5
Kraków	2548.1	3230.2	3297.2	8	9	10
Lublin	1302.3	1183.0	1380.6	9	8	9
Łódź	3318.2	3644.3	4044.6	11	12	13
Olsztyn	1893.0	2127.9	1851.3	22	23	20
Opole	362.8	643.0	585.1	4	6	4
Poznań	3516.9	3739.6	4475.9	13	13	16
Rzeszów	426.2	658.2	636.1	8	5	5
Szczecin	3030.8	3316.6	3497.0	10	10	11
Toruń	879.4	1008.9	1135.3	8	8	9
Warszawa	5129.7	4634.6	4988.3	10	8	9
Wrocław	2662.7	2930.6	3580.5	9	10	12
Zielona Góra	758.9	898.1	946.4	13	15	3 *

* The administrative area of the city was extended on 2 January 2015.

**Table 3:** The accessibility of green areas according to ANGSt standards in voivodeship cities in Poland.

City	All Households	Access to at Least 2 ha within 300 m [%]	Access to at Least 20 ha within 2 km [%]	Access to at Least 100 ha within 5 km [%]	Access to at Least 500 ha within 10 km [%]	All of the ANGSt Requirements Met [%]	None of the ANGSt Requirements Met [%]
Białystok	297,288	9	58	92	100	7	0
Bydgoszcz	352,313	20	48	89	100	17	0
Gdańsk	464,254	19	56	72	100	12	0
Gorzów Wlkp.	124,295	21	47	72	100	16	0
Katowice	281,950	12	45	68	100	5	0
Kielce	188,495	38	85	76	100	14	0
Kraków	76,748	26	60	79	100	12	0
Lublin	322,930	25	64	80	100	11	0
Łódź	687,702	18	56	78	100	10	0
Olsztyn	173,070	29	64	82	100	14	0
Opole	128,140	31	50	79	100	18	0
Poznań	671,233	20	62	76	100	12	0
Rzeszów	191,009	13	42	54	100	7	0
Szczecin	403,883	32	65	78	100	12	0
Toruń	182,725	21	54	62	100	10	0
Warszawa	1,764,615	20	67	72	100	6	0
Wrocław	638,600	22	66	87	100	8	0
Zielona Góra	139,819	12	62	85	100	10	0

**Table 4:** Comparison of the value of the green area indicator with the ratio of green areas to the city area according to the availability of at least 2 ha of green area at a distance of 300 m in provincial cities in Poland.

d	Access to at Least 2 ha within 300 m [%]	Indicator of Green Area per Inhabitant [m^2^/os]	The Ratio of Green Areas to the City’s Surface in 2018 [%]
Białystok	9	35.53	9
Katowice	12	30.42	5
Zielona Góra	12	68.02	3 *
Rzeszów	13	34.59	5
Łódź	18	58.2	13
Gdańsk	19	38.63	8
Warszawa	20	28.26	9
Bydgoszcz	20	54.44	10
Poznań	20	81.14	16
Gorzów Wlkp.	21	43.67	6
Toruń	21	56.92	9
Wrocław	22	56.65	12
Lublin	25	40.36	9
Kraków	26	42.97	10
Olsztyn	29	29.29	20
Opole	31	49.46	4
Szczecin	32	86.46	11
Kielce	38	30.28	5

* The administrative area of the city was extended on 2 January 2015.

**Table 5:** Comparison of access to green areas including spatial barriers.

Cities	Access to at Least 2 ha within 300 m [%]	Access to at Least 2 ha within 300 m with Spatial Barriers [%]	Differences Due to the Consideration of Barriers [%]
Białystok	6	5	−1
Katowice	12	8	−4
Zielona Góra	12	10	−2
Rzeszów	13	11	−2
Łódź	18	13	−5
Gdańsk	19	16	−3
Warszawa	20	11	−9
Bydgoszcz	20	17	−3
Poznań	20	16	−4
Gorzów Wlkp.	21	19	−2
Toruń	21	18	−3
Wrocław	22	17	−5
Lublin	25	22	−3
Kraków	26	20	−6
Olsztyn	29	26	−3
Opole	31	28	−3
Szczecin	32	29	−3
Kielce	38	35	−3
